# Luteolin peracetate and gossypolone inhibit immune complex-mediated neutrophil activation *in vitro* and dermal-epidermal separation in an *ex vivo* model of epidermolysis bullosa acquisita

**DOI:** 10.3389/fimmu.2023.1196116

**Published:** 2023-09-01

**Authors:** Kai Yang, Junping Yin, Xiaoyang Yue, Katja Bieber, Gabriela Riemekasten, Ralf J. Ludwig, Frank Petersen, Xinhua Yu

**Affiliations:** ^1^ Priority Area Chronic Lung Diseases, Research Center Borstel, Airway Research Center North (ARCN), Member of the German Center for Lung Research (DZL), Borstel, Germany; ^2^ Lübeck Institute of Experimental Dermatology and Center for Research on Inflammation of the Skin, University of Lübeck, Lübeck, Germany; ^3^ Department of Rheumatology and Clinical Immunology, University Clinic of Schleswig Holstein, University of Lübeck, Lübeck, Germany

**Keywords:** epidermolysis bullosa acquisita, neutrophils, natural products, luteolin peracetate, gossypolone

## Abstract

**Introduction:**

Natural products have been shown to an important source of therapeutics for human disease. In this study, we aimed to identify natural compounds as potential therapeutics for epidermolysis bullosa acquisita (EBA), an autoimmune disease caused by autoantibodies to type VII collagen (COL7).

**Methods:**

Utilizing an *in vitro* experimental system, we screened a natural product library composed of 800 pure compounds for their inhibitory effect on COL7-anti-COL7 IgG immune complex (IC)-mediated neutrophil activation and on neutrophil-mediated tissue damage.

**Results:**

Three natural compounds, namely luteolin peracetate, gossypol, and gossypolone were capable in inhibiting the IC-induced neutrophil adhesion and oxygen burst *in vitro*. Furthermore, luteolin peracetate and gossypolone were able to inhibit the anti-COL7 IgG induced dermal-epidermal separation in an *ex vivo* model for EBA.

**Discussion:**

In summary, this study demonstrates that luteolin peracetate and gossypolone are potential therapeutics for experimental EBA, which deserves further investigation.

## Introduction

Since more than two decades, Detlef Zillikens (†2022) and his group has worked on the fundamental pathological principles of Epidermolysis bullosa acquisita (EBA), a rare autoimmune skin blistering disease caused by autoantibodies targeting type VII collagen (COL7) (1, 2). It is characterized by subepithelial blistering of the skin at the dermal-epidermal junction (DEJ), a key structure to keep the integrity of the skin (3, 4). Anchoring fibrils, a main component of DEJ, is composed of COL7 and plays a crucial role in tethering the basal lamina to the underlying dermis function of DEJ (4). Once the circulating autoantibodies to COL7 migrate into the skin, the binding of those autoantibodies to the autoantigen at the DEJ is capable to mediate disease manifestations *via* inflammation dependent and independent pathways. In inflammatory EBA, binding of autoantibodies to COL7 leads to the formation of immune complexes (IC) which activate the complement system and mediate subsequent inflammatory cell infiltration leading to tissue destruction (5, 6). By contrast, non-inflammatory EBA is characterized by the lack of an autoantibody-mediated inflammatory responses and the development of skin symptoms appears to depend on non-inflammatory mechanisms (7, 8).

Accumulating evidence by Zillikens and others suggests that neutrophils play an essential role in the development of EBA and its experimental models (9–11). As a complicated process, neutrophil-mediated tissue damage consists of several essential steps, including recruitment of neutrophils into the skin and their binding to IC leading to cell activation and subsequent tissue damage (5, 6, 10, 11). It has been shown that oxygen radical formation and neutrophil degranulation are essential steps involved in the anti-COL7 IgG mediated tissue damage in experimental EBA (1, 11, 12). Previously, our group has demonstrated that anti-CD18 blocking antibodies could prevent the dermal-epidermal separation induced by anti-COL7 IgG, without affecting production of reactive oxygen species (ROS) or neutrophil elastase release (13). These findings clearly demonstrate that IC-induced neutrophil adhesion plays an indispensable role in tissue damage in experimental EBA, providing a potential novel target for the development of new therapies for the disease.

Natural products are defined as chemicals that are biosynthesized as secondary metabolites in various organism, including plants, animals, fungus as well as organisms from the marine environment (14, 15). Since the earliest record of natural products for treating diseases in the Ebers Papyrus (2900 B.E.C.), natural products have been proven to be the most prosperous and significant resource for the new medicines (16). Therefore, here we hypothesized that the tissue damage in experimental EBA can be attenuated by natural compounds which are capable of inhibiting IC-mediated neutrophils adhesion.

## Materials and methods

### Materials

The Natural product library was obtained from TimTec, Germany (NPL-800). The library contains 800 pure natural compounds, mainly of plant origin but also from animal, bacterial, and fungal sources. Rabbit anti-COL7 IgG was generated and purified as described previously (17). Immobilized immune complexes (IC) were formed from the binding of rabbit anti-mouse COL7 IgG to recombinant mouse COL7 pre-coated on the cell culture plate.

### Preparation of neutrophils and ethic approval

Neutrophils were isolated from peripheral blood of healthy donors by sedimentation and density gradient centrifugation as previously described (18). The purity and viability of neutrophils was determined by the morphological analysis of Jenner-Giemsa stained cytospins and Trypan Blue exclusion method, respectively (19). For all *in vitro* and *ex vivo* assays, neutrophils were cultured in CL medium (RPMI 1640 buffered with 25 mM HEPES without phenol red; Biochrom, Berlin, Germany) supplemented with 1% L-glutamine. This study was performed in accordance with the 1964 Helsinki Declaration, and the approval was obtained from the institutional ethics committee of the University of Lübeck (Number: Az 12-202A, from April 25, 2019). All donors agreed by written informed consent.

### IC-induced neutrophil adhesion

IC-induced neutrophil adhesion was measured by an electronic impedance-based assay using the xCELLigence^®^ Real Time Cell Analyzer (RTCA) which allows a real-time monitoring of the adhesion properties of cells *in vitro* in a non-invasive label-free manner (20, 21). Immobilized IC was prepared on wells of E-plate and freshly prepared neutrophils were added to wells in presence or absence of natural compounds. During the measurement, the electronic readout of cell sensor impedance was determined in real-time converted into cell index (CI). Neutrophil adhesion was quantified and expressed as the relative area under the curve (AUC) of cell index where the values of AUC of positive control (neutrophils stimulated with IC in absence of a compound) and negative control (unstimulated neutrophil) were set as 100% and 0%, respectively.

### Cell viability test

The cytotoxicity of natural compounds was determined by utilizing the Alamar Blue assay according to the protocol provided by the manufacture. Briefly, freshly isolated neutrophils at a final concentration of 1x10^6^/ml with a total volume of 150 μl in CL-medium supplemented with 1% L-glutamine were stimulated with immobilized IC in presence or absence of natural compounds on 96-well cell culture plates. Subsequently, 15 μl Alamar Blue reagent was added to each well, and spectrophotometric absorbance was determined every 60 mins for 7 hours. Cell viability was expressed as a relative value in which the value of positive control (unstimulated neutrophils) was set as 100%.

### IC-induced ROS release from neutrophils

To determinate IC-induced neutrophil ROS production, immobilized COL7-anti-COL7 IgG IC was prepared on solid opaque 96-well plates. Freshly isolated neutrophils were suspended in 2×10^6^/ml in CL medium supplemented with 1% L-glutamine and 5 μM luminol (5-Amino-2, 3-dihydro-1, 4-phthalazinedione; Roche Applied Science) and distributed in 200 µl aliquots into well microtiter plate coated with IC. After addition of neutrophils onto plates with immobilized immune complex, chemiluminescence was recorded by microplate luminometer (Berthhold Technologies GmbH, Germany) for 60 min in a real-time manner and data were expressed as relative light units (RLU).

### *Ex vivo* model of EBA

The *ex vivo* model of EBA was performed as previously described (17), with slight modification. Briefly, cryosections of mouse skin biopsies were incubated with rabbit anti-murine COL7 IgG, and further incubated with neutrophils in CL-medium supplemented with 1% L-glutamine to induce dermal-epidermal separation. Sections were washed in PBS, fixed with acetone acid, and subsequently stained with hematoxylin and eosin. Skin dermal-epidermal separation was evaluated by light microscopy, and the extent of dermal-epidermal separation was analyzed in a blinded fashion by two persons. This study was approved by the Animal Research Ethics Board of the Ministry of Environment, Kiel, Germany.

### Statistical analysis

All data are expressed as the mean ± SD (standard deviation) or mean ± SEM (standard error of mean). Quantitative data was firstly examined with Kolmogorov-Smirnov normality test. For the normally distributed data, statistical difference was determined by two-tailed paired Student’s t test, otherwise Wilcoxon signed rank test was used. For the data with two or more independent variables, statistical analysis was performed with two-way ANOVA test. For the evaluation of dose response relationship, linear regression analysis was performed, and the F-test was applied to assess the statistical significance. P values less than 0.05 were considered as statistically significant.

## Results

### Blocking of IC-induced neutrophil adhesion

**Figure 1:**
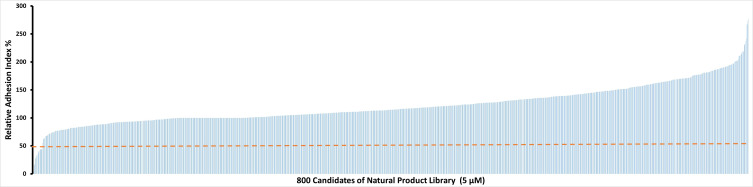
Effects of 800 natural products on IC-mediated neutrophil adhesion. Freshly isolated human neutrophils (2×10^6^/ml) were stimulated with immobilized COL7-anti-COL7 IgG IC in the presence or absence of a natural product at a concentration of 5 μM. Neutrophil adhesion was determined by real-time impedance measurement for 90 minutes using xCELLigence RTCA and expressed as relative adhesion index where the value of AUC of positive control (neutrophils stimulated with IC in absence of the compound) and negative control (unstimulated neutrophils) were set as 100% and 0%, respectively. The X-axis stands for the 800 compounds of natural product library, and the Y-axis presents the mean relative adhesion index of two independent experiments. The dashed line represents 50% adhesion index of the positive control.

To identify potential candidate compounds that are able to inhibit IC-induced neutrophil adhesion, the Natural Products Library which contains 800 compounds was screened. Among the 800 tested natural products, 11 showed relative AUC values of lower than 50% (**Figure 1**). These 11 natural compounds were luteolin peracetate, luteolin, capsaicin, gossypol, gossypolone, digitonin, plumbagin, estradiol valerate, 2-Chloroadenosine, 3,3-Diindolylmethane and peracetate rutine. In addition, a few molecules showed relative AUC values of higher than 250%, including digoxigenin, sinomenine and emetine dihydrochloride hydrate.

**Figure 2:**
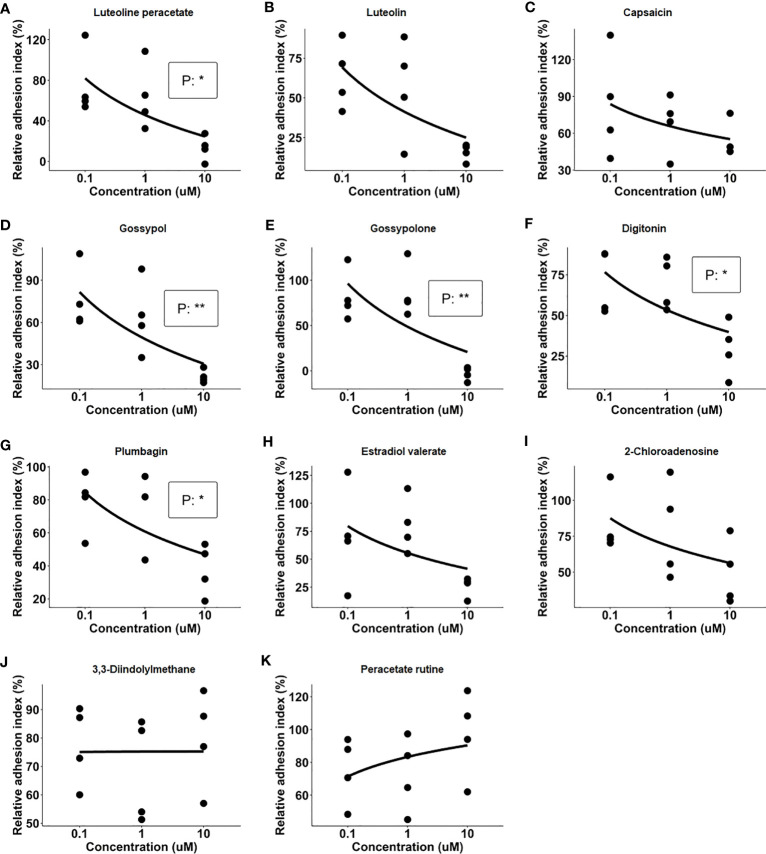
Dose-response relationships of 11 natural products. Freshly isolated neutrophils were stimulated with immobilized immune complex (IC) on wells of E-plate in presence of indicated concentrations of candidate compounds, including Luteolin peracetate **(A)**, Luteolin **(B)**, Capsaicin **(C)**, Gossypol **(D)**, Gossypolone **(E)**, Digitonin **(F)**, Plumbagin **(G)**, Estradiol valerate **(H)**, 2- Chloroadenosine **(I)**, 3,3-Diindolylmethane **(J)**, and Peracetate Rutine **(K)**. Cell indexes were recorded for 90 minutes, and AUC of Delta Cell Indexes was calculated. For further analysis, neutrophil adhesion was expressed as relative adhesion index where the value of AUC of positive control (neutrophils stimulated with IC in absence of the compound) and negative control (unstimulated neutrophils) were set as 100% and 0%, respectively. The X-axis presents doses of natural products, while Y-axis shows the relative adhesion indexes. Data from four independent experiments were used for analysis. Linear regression analysis was performed to evaluate the dose-response relationship, and the statistical significance was assessed using the F-test (*p<0.05, **p<0.01).

After the primary screening, the 11 natural products which showed an inhibitory effect on IC-mediated neutrophil adhesion were selected for further evaluation. In this part, dose-dependency on the inhibition of neutrophil adhesion by the selected 11 natural products was determined in a range between 0.1 µM and 10 µM of the respective compound. The dose-response assay was performed in four independent experiments by using the xCELLigence RTCA system with neutrophils isolated from 4 healthy donors, respectively. Among the 11 tested natural products, 5 products, namely luteolin peracetate, plumbagin, digitonin, gossypol, and gossypolone showed significant dose-response relationships (**Figure 2**).

### Cytotoxicity of compounds

**Figure 3:**
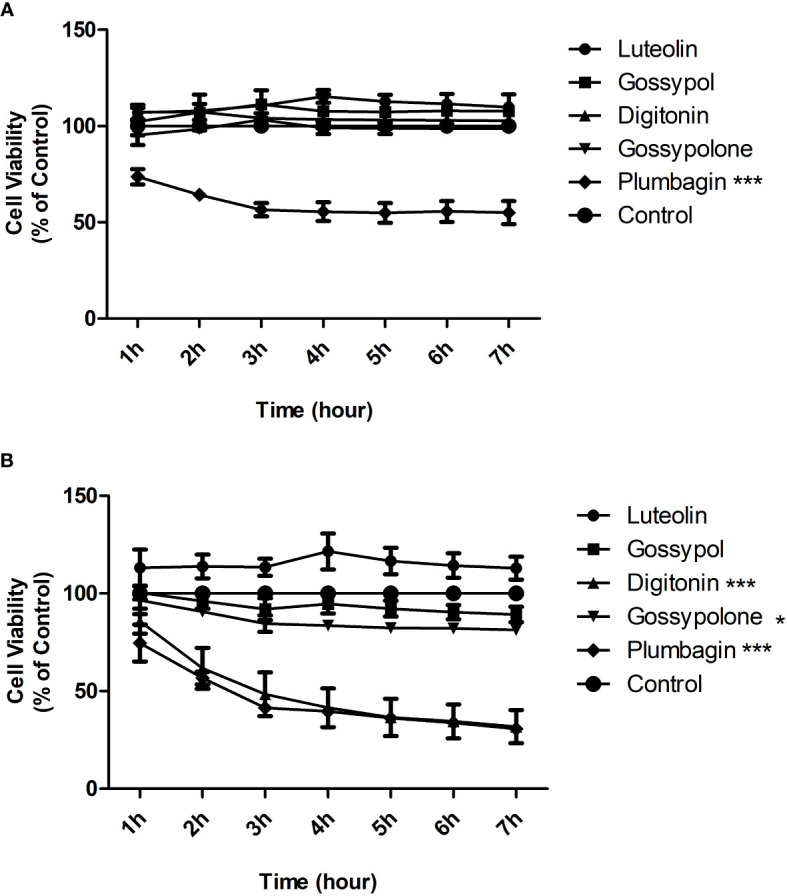
Cytotoxicity effects of 5 natural products on neutrophils. Freshly isolated human peripheral blood neutrophils were stimulated by immobilized IC in presence or absence (control) of indicated natural product at a concentration of 1 μM **(A)** or 10 μM **(B)**. Cell viability was assessed by using the Alamar Blue cytotoxicity assay. The Alamar Blue was added 1 hour after the cell incubation, and the absorbance was recorded every hour for 6 hours. Data are presented as mean ± SEM (standard error of the mean) of 4 independent experiments. Cell viability was expressed as a relative value where the value of control (unstimulated neutrophils) was set as 100%. The toxic effect of each natural product was evaluated by comparing the cell viability between product-treated neutrophils and controls using two-way ANOVA test (*p<0.05, ***p<0.001).

To exclude that inhibition on neutrophil adhesion is a consequence of toxic effects, toxicity test was carried out for the five natural products. Toxicity was evaluated by determining the cell viability using the Alamar Blue cytotoxicity assay. Neutrophils were stimulated with immobilized IC in the presence or absence of candidate natural products at a concentration of 1 μM or 10 μM, and Alamar Blue was added to cells 1 hour later to monitor the cell viability for 6 hours. At the concentration of 1 μM, plumbagin showed a strong and significant toxic effect on neutrophils, with a decrease of 50 percent in cell viability after 3h of incubation (**Figure 3A**). At the concentration of 10 μM, significant toxic effects were observed for both plumbagin and digitonin, with decreases of 60 percent in cell viability for both products after 3h of incubation (**Figure 3B**). In addition, Gossypolone at the concentration of 10 μM also showed a significant but mild toxic effect on neutrophils, with a decrease of roughly 10 percent in cell viability after 3h of incubation, but such effect was not observed at the concentration of 1 μM (**Figure 3A**). Therefore, luteolin peracetate, gossypol, and gossypolone were selected for further investigation.

### Blocking of neutrophils ROS production

**Figure 4:**
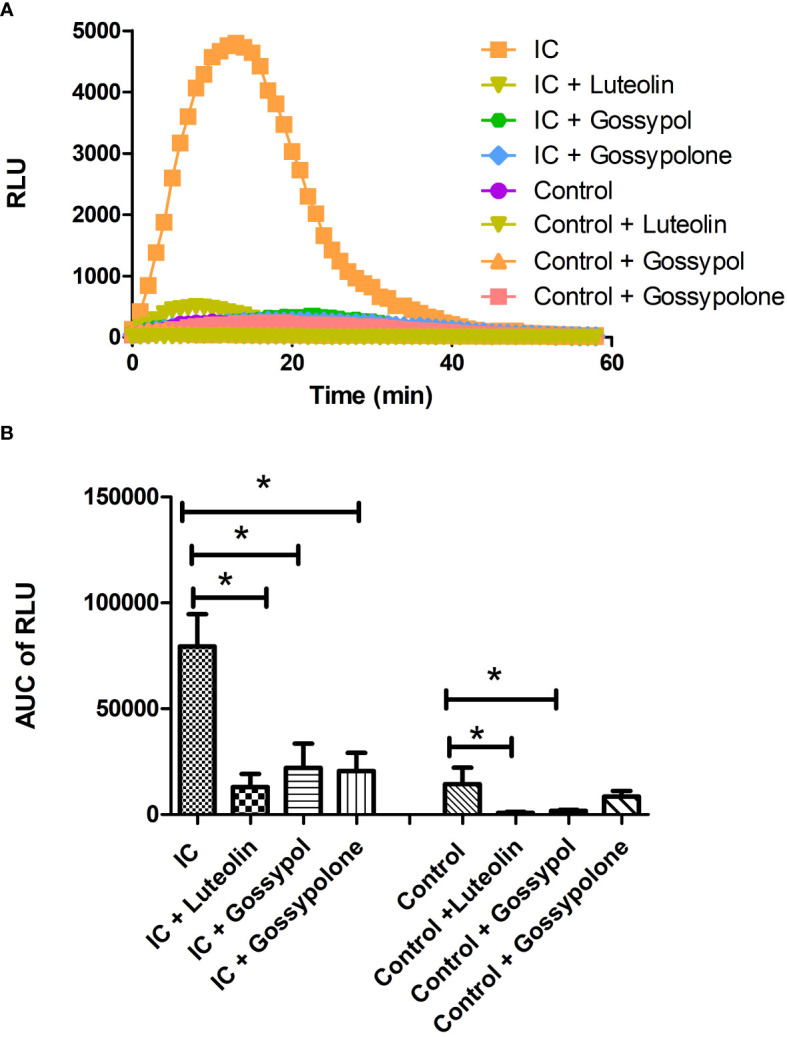
Effect of selected natural products on IC-induced ROS production of neutrophils. Freshly neutrophils were stimulated with immobilized IC or control in the presence or absence of a natural product at the concentration of 10 μM. Generation of ROS was determined by recording chemiluminescence using microplate luminometer for 60 min in a real-time manner and data were expressed as relative light units (RLU). **(A)** Representative kinetics of ROS production from neutrophils under indicated experimental conditions. **(B)** Kinetic data of IC-mediated ROS production from neutrophils were integrated and represented as the AUC (area under the curve) of RLU (relative light units). Data are presented as mean ± SD derived from 6 experiments, and statistical analysis was performed using Wilcoxon signed rank test (*p<0.05).

To figure out the effects of the 3 selected candidates on ROS production of neutrophil, freshly isolated neutrophils were stimulated with immobilized IC or control in presence or absence of the respective candidate compounds at a concentration of 10 µM. As shown in **Figure 4A**, time kinetics of ROS production showed that neutrophils immediately produced ROS when they attached to immobilized IC. The produced ROS reached a peak at approximately 10 minutes and returned to background level after 40 minutes. In contrast, neutrophils exposed to control surfaces did not show a significant ROS production. When neutrophils were exposed to immobilized IC in the presence of luteolin peracetate, gossypol or gossypolone, the IC-mediated ROS production was significantly decreased. Quantification of the ROS production in 6 experiments showed that all three natural products significantly inhibited the IC-induced ROS production from neutrophils (**Figure 4B**).

### Inhibition of dermal-epidermal separation in an ex vivo model of EBA

**Figure 5:**
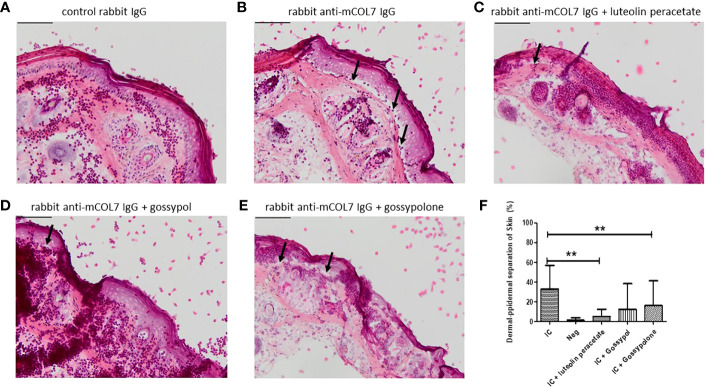
Effect of luteolin peracetate, gossypol and gossypolone on tissue damage in experimental EBA ex vivo. Mouse skin cryosections were incubated with 0.2 mg/ml rabbit control IgG **(A)** or rabbit-anti-mouse COL7 IgG **(B-E)** for 1 hour at 37°C. Subsequently, specimens were exposed to freshly isolated human neutrophils **(A, B)** or neutrophils in presence of 0.1% DMSO **(B)**, 10 μM luteolin peracetate **(C)**, 10 μM gossypol **(D)** or 10 μM gossypolone **(E)**. Images of the skin section were acquired using the NIS-Element D 3.0 software and an OLYMPUS Bx41 microscope. Micrographs of a representative experiment are shown, and black arrows indicate the dermal-epidermal separation. Scale bar = 100 µm. Skin separation was quantified as percentage of the length of epidermis detachment in relation to the length of the total dermal-epidermal zone **(F)**. Data are presented as mean ± SD of 9 independent experiments. Statically significant differences were performed using Wilcoxon signed rank test (**p<0.01).

Finally, we determined whether the three natural compounds are able to inhibit anti-COL7 IgG mediated tissue damage in an *ex vivo* model for EBA. In the experimental system, neutrophils activated by the COL7-anti-COL7 IC caused the dermal-epidermal separation of 33 ± 7.9%, while no separation was observed in the negative control in which anti-COL7 IgG was replaced by normal rabbit IgG (**Figure 5**). In the presence of 10 μM luteolin peracetate, the dermal-epidermal separation was significantly reduced to 4.9 ± 2.6% (P = 0.009). Significant and dramatic reduction in dermal-epidermal separation was also observed in the samples treated with 10 μM gossypolone (16.1 ± 8.4%, P=0.0091). In the presence of 10 μM gossypol, although the dermal-epidermal separation was also reduced to 12.5 ± 8.6%, this difference was not significant (P=0.0547). Taken together, these results suggest that both luteolin peracetate and gossypolone can inhibit tissue damage in the ex vivo model for EBA.

## Discussion

In the present study, we screened a natural product library of 800 pure compounds for their inhibitory capability on anti-COL7 IgG mediated tissue damage. We demonstrated that two compounds, luteolin peracetate and gossypolone, showed a strong and significant inhibitory effect on anti-COL7 IgG mediated tissue damage in an ex vivo model for EBA. Two compounds, luteolin peracetate and gossypolone, showed a strong and significant inhibitory effect on IC-induced neutrophil tissue damage. Importantly, both luteolin peracetate and gossypolone did not only inhibit the IC-mediated neutrophil adhesion, but also blocked IC-mediated ROS production of neutrophils, suggesting that both natural products represent inhibitors for neutrophil functions in general.

Luteolin peracetate is a flavonoid, with a typical structures with C6-C3-C6 (22). Although it is well established that flavonoids are featured by pharmacological properties of anti-inflammatory, immunomodulatory functions, antioxidant, anti-cancer and antimicrobial effects (23, 24), to our best knowledge the pharmacological property of luteolin peracetate has not been described so far. By contrast, luteolin, a flavonoid highly related to luteolin peracetate, has been well studied in the past (25). As one of the most common flavonoids, luteolin are found in around 300 plant species, including many edible plants, such as celery, broccoli, green pepper, parsley, thyme, dandelion, perilla, chamomile tea, carrots, olive oil, peppermint, rosemary, navel oranges, and oregano (25, 26). The current study showed that luteolin peracetate could inhibit neutrophil adhesion induced by immobilized IC. It has been reported that multiple flavonoids inhibit neutrophils migration both *in vitro* and *in vivo* by acting on multiple mechanisms, including decreasing the expression of β2-integrin expression of neutrophils (27). In addition, it has been demonstrated that the IC-induced neutrophil adhesion is dependent on β2-integrin (13). Therefore, luteolin might inhibit the IC-induced neutrophils adhesion and subsequent tissue damage by inhibiting the β2-integrin expression on neutrophils. However, this hypothesis needs to be validated in future studies. Besides its capacity to inhibit IC-induced neutrophil adhesion, luteolin peracetate was able to block anti-COL7 IgG-mediated tissue damage *via* decreasing IC-mediated neutrophil oxygen burst. In the current study, luteolin peracetate at the concentration of 10 μM significantly and considerably reduce the IC-induced production of ROS in neutrophils. This finding is in line with the result from a previously study, where Oswald et al. reported that luteolin, a flavonoid highly related to luteolin peracetate, is able to suppress the ROS production from neutrophils stimulated with COL17-anti-COL17 IC in a dose-dependent manner (28).

In the *ex vivo* model for EBA used in this study, gossypolone inhibited significantly anti-COL7-induced tissue damage. These results suggest that gossypol and its derivatives could be potential therapeutics for EBA. As a natural occurring polyphenol, gossypol is isolated from the seed, roots, and stem of cotton (29). Due to its antifertility properties, it has long been known as a male contraceptive. Moreover, several biological effects such as anticancer, antivirus, antiparasitic and antimicrobial activities have been uncovered recently, making, Gossypol interesting as a potential drug for several cancer and chronic infectious diseases (29–31). By contrast, effects of gossypol and gossypolone on inflammation are much less extensively investigated. By administering gossypol as one potential antioxidant inhibitor orally and intrarectally, Fitzpatrick et al. discovered the anti-inflammatory activity of gossypol in an *in vivo* model of colitis around 30 years ago (32). Regarding the mechanism underlying the anti-inflammatory effect, an inhibition of lymphocyte proliferation, a suppression of inflammatory cytokines, and an antioxidative effect have been suggested (33–35). In the present study, gossypol and gossypolone are demonstrated to be able to inhibit IC-induced neutrophil adhesion and ROS production which is in line with results from previous studies. For example, Wang and colleagues showed that the hydroxyl groups of gossypol play a significant role in scavenging free radicals in various tumor cell lines (36). In addition, it has been reported that gossypol and gossypolone show antioxidant activity and have been suggested as potential therapeutics for psoriasis (33). By contrast, Benhaim et al. reported that pretreatment of gossypol could induce the superoxide production of human neutrophils in a time- and concentration-dependent manner, suggesting a pro-oxidant activity of gossypol (37). Therefore, the effect of gossypol and its derivatives on neutrophil ROS production might be condition-dependent, where they show pro-oxidant effects on unstimulated neutrophils and antioxidant effects on neutrophil activated by other stimuli.

## Conclusion

In this study, we could demonstrate that luteolin peracetate and gossypolone are able to inhibit the anit-COL7 IgG-mediated tissue damage, providing novel potential therapeutics for EBA. However, the safety and efficacy of these compounds need to be further evaluated in animal studies *in vivo*. In addition, given shared mechanisms between EBA and bullous pemphigoid (BP), another autoimmune skin blistering disease caused by autoantibodies against COL17 (38, 39), it is conceivable to determine the effect of the two natural compounds on experimental BP.

This work has only been possible because of Prof. Dr. Detlef Zillikens. With his full support, in 2008 we started to our research in the field of autoimmune skin blistering diseases at the Research Center Borstel, Germany. We truly miss him as a motivating and supportive cooperation partner and friend.

## Data availability statement

The original contributions presented in the study are included in the article/supplementary material. Further inquiries can be directed to the corresponding author.

## Author contributions

XYu and FP conceived the study. XYu, FP and GR supervised the study. KY, JY, XYue and KB performed the experiments and analyzed the data. RL provided essential materials and methods. KY and XYu wrote the manuscript and all authors revised the manuscript. All authors contributed to the article and approved the submitted version.
